# Effects of Sex and Hunting Season on Carcass and Meat Quality Characteristics of the Brown Hare (*Lepus europaeus*)

**DOI:** 10.3390/foods12122369

**Published:** 2023-06-14

**Authors:** Violeta Razmaitė, Artūras Šiukščius

**Affiliations:** Department of Animal Breeding and Reproduction, Animal Science Institute, Lithuanian University of Health Sciences, R. Žebenkos 12, 82317 Baisogala, Lithuania; arturas.siukscius@lsmu.lt

**Keywords:** game, meat, fat, fatty acids, hare, carcass

## Abstract

The objective of the study was to determine the effects of sex and hunting season on the carcass, meat and fat quality of hunted brown hares (*Lepus europaeus*). Twenty-two hares of both sexes hunted in winter (December) during two hunting seasons in accordance with the law on hunting in Lithuania were evaluated using reference methods The data were subjected to two-factor analysis of variance in the general linear (GLM) procedure. No significant differences in carcass measurements and muscularity or internal organs between the sexes of brown hares were found; however, the hunting season appeared to affect the size of hares. The *biceps femoris* (BF) thigh muscle of males had lower (*p* < 0.05) dry matter content and higher (*p* < 0.05) drip loss compared with females. The hunting season demonstrated an effect (*p* < 0.001) on protein and hydroxyproline contents in the *longissimus thoracis et lumborum* (LTL) and affected dry matter, protein and hydroxyproline contents (*p* < 0.05, *p* < 0.001 and *p* < 0.01, respectively) in BF muscles, and differences in the colour of muscles were also observed. The shear force in the Warner–Bratzler (WB) test was higher (*p* < 0.001 and *p* < 0.01, respectively) for LTL and BF muscles during the first hunting season. The hunting season did not affect the total SFA in the intramuscular fat (IMF) of all the tissues, but it affected levels of monounsaturated (MUFA) and polyunsaturated (PUFA) fatty acids in the muscles. No differences were found in the total saturated fatty acids (SFA) of both muscles between the sexes, but females demonstrated lower (*p* < 0.05 and *p* < 0.01, respectively) and more favourable n-6/n-3 PUFA ratios in the muscles and fat and a lower (*p* < 0.05) thrombogenic (TI) index in the LTL compared with males.

## 1. Introduction

Meat supplies nutrients and plays a vital role in human life, being a crucial component of a well-balanced diet [1]. Despite its nutritional richness, meat consumption has been considered a disease-promoting food [1,2,3,4,5]. Although different studies support the opinion that high meat intake may increase the risk of cancer, it is important to recognise that meat is an important source of nutrients, several of which have potential anticancer properties [6], and that there are systems for producing safer meat [4,5]. The concept of meat quality includes both eating and technological quality, nutritional value and safety, and meat diversification is also a quality. Consumers are also increasingly concerned about healthy products and show interest in the consumption of meat from alternative species whose meat is seen to be healthy, thus combining the requirements of consumers who are attentive both to gourmet food and to their own health [7,8,9]. Therefore, the demand for alternative meats, and particularly meat from wild animals, is increasing. The brown hare (*Lepus europaeus*) is common throughout almost all of Europe [10,11,12] and has been introduced to different countries in other continents [12,13]. The European or the brown hare (*Lepus europaeus*) has been recognized as an important game species throughout its historical distribution, including in those areas where it has been introduced [11,14]. As a valuable game species, hares are among the wild mammals that are used widely in many recipes [15,16].

The brown hare (*Lepus europaeus*) is one of two hare species in Lithuania, the territory of which is the southern edge of the distribution area of another species, *Lepus timidus*. *Lepus europaeus* was rarer than *Lepus timidus* during the 15th–18th centuries in Lithuania, but later, the brown hare became dominant [17]. According to Bluzma [18], the most abundant population of *Lepus europaeus* in Lithuania was in the 1970s, but since the 1960s the population of *Lepus europaeus* in many European countries has been declining as a result of multiple factors [12,19,20,21,22,23], and therefore the need to regulate their populations has arisen [12,23]. Since 1989, hunting of *Lepus timidus* has been prohibited [24], and currently in Lithuania, only brown hares (*Lepus europaeus*) are hunted at a moderate intensity. The annual hare abundance fluctuates by 5–10% [25]. As it is possible to keep *Lepus europaeus* in captivity for research purposes [11,26,27], in some countries, brown hares have started to be farmed to restore their local populations [28] and for restocking purposes, thus offering additional game [29,30].

Certain results have been published regarding the meat quality of the brown hare [31], but many of these studies present data on the farmed hare [26,27,28,30,32]. Information is still missing about the carcass and meat quality of free-living brown hares, particularly about the influence of their sex and hunting seasonality on the carcass and meat characteristics. However, the quality of the game could be also influenced by the geographical area of the hunt. Information on the nutritional value of hare fat is also scarce. Therefore, the objective of the study was to determine the effects of sex and season on the carcass, meat and fat quality of hunted brown hares (*Lepus europaeus*).

## 2. Materials and Methods

### 2.1. Animals and Sampling

The brown hare used in this study was shot in accordance with the hunting law of the Republic of Lithuania; namely, law No IX-966 of 18 June 2013. Twenty-two hares (11 males and 11 females) hunted in winter (December) during two hunting seasons in the forests of the northern part of Lithuania at a latitude of 55°33′ to 56°18′ N and longitude of 22°30′ to 24°07′ E were used in the experiment. The skin, fore and hind distal legs, gastrointestinal tract and perirenal fat were removed in a special area for dressing hunted animals. The eviscerated carcasses were transported to the laboratory and stored at 4 °C until evaluation within 24 h after the shooting.

### 2.2. Carcass Evaluation and Meat Sampling

The chilled carcasses with the head and the liver but without the gall bladder, lungs, heart and kidneys were weighed to obtain the carcass weight. Measurements of the carcass length and lumbar circumference were taken with a tape. The dorsal length was measured as the interval between the atlas vertebra and the 7th lumbar vertebra. The thigh length was measured as the interval between the 7th lumbar vertebra and the distal part of the os ischii. Lumbar circumference was measured at the level of the 7th lumbar vertebra [33]. The left hind legs were separated to measure their weight, and the meat was separated from the bones to measure the meat-to-bone ratio as an indicator of carcass muscularity [33]. For further meat and lipid analysis, the samples of the two muscles of different carcass sites—*m*. *longissimus thoracis et lumborum* (LTL), *m. biceps femoris* (BF; hind quarter)—and scapular fat were used.

### 2.3. Meat Quality Assessments

#### 2.3.1. Chemical Composition of Muscles

The dry matter content was determined [34] by drying samples in an oven at 105 °C until a constant weight was obtained (method No. 950.46B) in [34]. The crude protein content was determined by the Kjeldahl method using the Kjeltec system 1002 apparatus (Foss-Tecator AB, Höganäs, Sweden), and a conversion factor of 6.25 was used to convert total nitrogen to crude protein (method No. 981.10) in [34]. Crude fat was determined by the Soxhlet extraction method (method No. 960.39) in [34]. Ash was determined by incineration in a muffle furnace at 550 °C for 24 h (method No. 920.153) in [34]. The content of protein, fat and ash was expressed as the weight percentage of dry matter from muscle tissues. The hydroxyproline content was determined by the NMKL-AOAC method [35].

The cholesterol content in meat was determined according to the extraction method described by Polak et al. [36], followed by HPLC separation and analysis on a Shimadzu 10 A HPLC system (Shimadzu Corp., Kyoto, Japan). The data collection and evaluation were performed by using the LC Solution (Shimadzu Corp., Kyoto, Japan) operating system. The analytical column was LiChrospher 100 RP-18e, 150 × 4.6 mm, 5 μm (Alltech Associates Inc., Chicago, IL, USA) with a guard column (LiChrospher 100 RP-18, 7.5 × 4.6 mm). The cholesterol content was expressed as mg/100 g of fresh meat.

#### 2.3.2. pH Measurements

The pH value was measured 48 h post-mortem using the PT-380 digital portable pH-meter (Boeco, Hamburg, Germany) equipped with a glass electrode (Witeg Laboratory Technik GMBH, Wertheim, Germany) after calibration using pH 4.0 and 7.0 buffer solutions.

#### 2.3.3. Colour

The colour parameters were measured using the CR-410 Konica Minolta (Tokyo, Japan) chromameter. The device specification, calibration and measurements were described in the previous study [37].

#### 2.3.4. Water Holding Capacity

Water holding capacity was measured in two ways: drip loss and cooking loss. The drip loss was assessed according to the EZ-DripLoss method [38]. The cooking loss was determined as described in the previous study [37,39].

#### 2.3.5. Instrumental Evaluation of Texture

The tenderness of the *longissimus thoracis et lumborum* and *biceps femoris* muscles was instrumentally measured by the Warner–Bratzler shear test (WB) and by texture profile analysis (TPA) using a Texture Analyser TA 1 (Measurement and Calibration Technologies Ametek Comp., Lloyd instruments, Largo, FL, USA) as described in the previous study [37]. 

### 2.4. Fatty Acid Profiles

The extraction of lipids for fatty acid analysis was performed as described by Folch et al. [40]. Fatty-acid methyl esters were prepared according to the procedure described by Chistopherson and Glass [41]. Methylation of the samples and FAMEs analysis were performed as described in the previous study [37] using a gas–liquid chromatograph (GC–2010 SHIMADZU, Kyoto, Japan) fitted with a flame ionization detector. The peaks were identified by comparison with the retention times of the standard fatty-acid methyl esters “37 Component FAME Mix” and “trans FAME MIX k 110” (Supelco, Bellefonte, PA, USA). The relative proportion of each fatty acid was expressed as the relative percentage of the sum of the total fatty acids using the “GC solution” software for Shimadzu gas-chromatograph workstations.

### 2.5. Lipid Quality Indices

Lipid quality indices, i.e., the atherogenic index (AI) and the thrombogenic index (TI), were calculated according to Ulbricht and Southgate [42]. The hypocholesterolemic/hypercholesterolemic (h/H) ratio was calculated according to Santos-Silva et al. [43]. The peroxidizability index (PI) was determined according to Du et al. [44].

### 2.6. Statistical Analysis

The data were subjected to two-factor analysis of variance in a general linear (GLM) procedure in IBM SPSS Statistics 27 (IBM, Armonk, NY, USA) with LSD tests to determine the significance of differences in estimated marginal (EM) means between the sexes and seasons. The GLM model included fixed factors of sex and hunting season as well as factor interactions (sex × season). The differences were regarded as significant when *p* < 0.05, but differences of 0.05 ≤ *p* < 0.10 were considered trends.

## 3. Results and Discussion

### 3.1. Carcass and Internal Organs

The hunting season appeared to affect the size of brown hares. The hares shot during the second hunting season showed higher (*p* < 0.01 and *p* < 0.05, respectively) carcass weight and dorsal length compared with the previous season (Table 1). The different sizes of shot hares in individual seasons may have been caused by uncontrolled environmental and nutritional conditions and by blind chance during their hunt. Only slight variations was found in these and other carcass measures, such as lumbar circumference and muscularity, in terms of the meat-to-bone ratio in the thighs of brown hares between males and females; this is consistent with the findings of other authors [26,45] and confirms the lack of external sexual dimorphism in the brown hare [46]. No significant interactions were found between the sex and hunting season. It is difficult to compare the carcass weights indicated in the present study with the carcass weight of the hares hunted in other countries. In this study, the weight of skinned and eviscerated carcasses was determined with internal organs but without the perirenal fat present, while other authors analysed the carcass weight without the head and internal organs [45] or only of skinned and eviscerated carcasses [47]. In fact, the carcasses of the hares hunted in Lithuania are quite large. More details of carcass evaluation have been reported on hares bred in captivity where feeding, age and slaughter conditions could be controlled [26,30]. The overall thigh proportion in the carcass (32.05%) obtained in the present study was lower than those estimated in farmed hares, whose hind leg proportions accounted for 36.3% [30] and 38.5% [26]. Moreover, the meat/bone ratio in the present study was also lower and showed lower muscularity compared with farmed hares [30].

There were no significant differences between the sexes regarding the internal organs of hares hunted in winter (Table 2).

The weight of the organs, except of the kidneys, was similar to the weight of the corresponding organs of hares hunted in Croatia [47] and higher than the weight of the organs of hares hunted in Poland [45], and this is an indication that the geographical area does not affect either the internal organs or the sex.

### 3.2. Meat Quality

The meat chemical composition displayed in Table 3 did not show any significant differences between the sexes of brown hare in the *longissimus thoracis et lumborum* (LTL) muscles, but the *biceps femoris* (BF) thigh muscles of males had lower (*p* < 0.05) dry matter content compared with females. The LTL muscle [48] and the triceps brachii muscle [49] of brown hare males shot in Slovakia and Romania had a higher and lower content of protein and fat, respectively, compared with females. The same pattern for intramuscular fat (IMF) content could be seen in the present study; however, the differences in most of the parameters of meat chemical composition between the sexes were insignificant. Farmed hare males [30], conversely, had more fat in the hind leg than females. Neither the muscle nor the sex showed any significant differences in the cholesterol content; however, the cholesterol content detected in the present study was considerably lower than that of the farmed hare [27]. Therefore, in terms of cholesterol content, meat from hares living in freedom can be considered more valuable than that from farmed ones. Nevertheless, as our previous research showed [50], hare meat can be characterized by a higher cholesterol content compared with other different animals of meat-producing species. 

Meat differences most probably depend on the species, and considering the animal’s diet is important in quantifying the differences in the composition and quality of meat derived from different muscles. Due to different muscle anatomical locations and functions, including the physical activity level of skeletal muscles, the physicochemical composition of muscles differs and, accordingly, influences the differences in the characteristics of meat [51]. The hunting season demonstrated an effect (*p* < 0.001) on protein and hydroxyproline contents in the LTL and effects (*p* < 0.05, *p* < 0.001 and *p* < 0.01, respectively) on dry matter, protein and hydroxyproline contents in BF muscles. Lithuanian hare meat can be characterized as having less protein and more fat content than the meat of hares shot in more southern areas such as Slovakia [48] and Croatia [47].

The sex did not show any effect on most physical traits of wild brown hare LTL muscles, while the sex affected the drip loss of the BF thigh muscle. Males showed a higher (*p* < 0.05) drip loss compared with females (Table 4). In the present study, meat from brown hares hunted during their peak mating season—which, in Lithuania, mostly occurs in January—was not analysed, and as no literature data was found about the influence of hare sexual activity on meat quality, this study neither confirms nor denies that the absence of sex effect or a limited sex effect depends on whether hares were hunted before they began to mate. Mertin et al. [48] have reported higher meat lightness of wild hare males compared with females. The hunting season, in contrast to sex, appeared to affect the meat quality of brown hares. Both LTL and BF muscles showed higher (*p* < 0.001) pH, lightness (L*) and drip loss and lower (*p* < 0.001) muscle yellowness (b*) and hue (h) for the hares hunted during the second hunting season compared with the previous season. The cooking loss was also higher (*p* < 0.05 and *p* = 0.051, respectively) in the LTL and tended to be higher in BF muscles from the hares hunted during the second hunting season when the hunted hares were larger. 

### 3.3. Meat Texture

The results of the hare meat texture profile analysis (TPA) and the Warner–Bratzler (WB) test did not show significant differences in both the LTL and BF muscles between the sexes of the wild brown hare (Table 5). However, cohesiveness of the BF muscle appeared to be higher (*p* < 0.05) during the first hunting season. The shear force in the WB test was higher (*p* < 0.001 and *p* < 0.01, respectively) for both LTL and BF muscles during the first hunting season, while higher (*p* < 0.001) WB toughness was measured only for the BF muscle.

### 3.4. Fatty Acid Profiles

There were no differences found in the percentage of total saturated fatty acids (SFA), including individual saturated fatty acids, in both LTL and BF muscles between the sexes of the wild brown hare (Table 6). 

In their review, Neethling et al. [51] noted that meat from female game animals contains significantly higher amounts of fat compared with males and, consequently, also contains higher proportions of SFA; however, no such relation was found for the brown hare. A high amount of palmitic (C16:0) fatty acid in the tissues of the brown hare is in agreement with the findings for wild hares in Romania [52]. Similarly to the present study, there were also no differences found in the SFA between the muscles from different carcass parts of farmed brown hares [32]. However, SFA contents reported in other different studies [26,30,32] for muscles of the farmed hare were higher than in the present study for wild hares hunted in winter. Although the hunting season did not affect the total SFA, it did have an effect (*p* < 0.01, *p* < 0.05 and *p* < 0.001, respectively) on individual myristic (C14:0), margaric (C17:0) and stearic (C18:0) fatty acids in the LTL muscle and also affected (*p* < 0.01 and *p* < 0.05) the same C14:0 and C18:0 acids and the arachidic (C20:0) fatty acid in the BF muscle. It is likely that in different seasons and years, wild hares eat different types of forage, even in the same geographical area. The locomotor performance or running speed should be associated with differences in the muscle fatty acid profiles of such an extremely fast runner as the brown hare [50] that lives in the temperate and cold areas of Europe without shelter throughout the year and that requires energy intake for maintenance and growth, higher than that of other mammals that live in nests or burrows [11,53,54]. Schai-Braun et al. [54] have reported that hares select their diet for high crude fat, including PUFA and crude protein, and they avoid crude fibre. Therefore, they prefer weeds/grasses and various crop types having more fat and avoid cereals, and they are highly selective in their food choice during winter. On the other hand, hares use the feed that is available. In Lithuania, the eating habits [17] of brown hares are similar, but under harsh winter conditions, the diet of hares also includes the bark of young trees and shrubs; however, they avoid the bark of the alder tree. Their favourite is the bark of young fruit trees.

There were no differences in the percentages of total monounsaturated fatty acids (MUFA) in both LTL and BF muscles between the sexes of the wild brown hare (Table 7). A higher (*p* < 0.05) amount of elaidic (C18:1trans-9) fatty acid was detected in the intramuscular fat of the LTL muscle of females compared with males. However, the hunting season affected (*p* < 0.001 and *p* < 0.01, respectively) the total MUFA, including the most abundant individual oleic (C18:1n-9) fatty acid, in both the LTL and BF muscles.

Additionally, the hunting season showed an effect (*p* < 0.05) on the C15:1 and C16:1n-7 acids in the LTL and on the C16-1n-9 acid in BF muscles. 

Although the sex of the wild hare did not affect the content of total of polyunsaturated fatty acids (PUFA) in the muscles, the composition of PUFA exhibited the largest differences between the sexes of the brown hare and, particularly, between their hunting seasons (Table 8). Females showed higher (*p* < 0.05) percentages of individual α-linolenic (C18:3n-3) and eicosatrienoic (C20:3n-3) fatty acids in LTL muscle, and higher (*p* < 0.01 and *p* < 0.05, respectively) percentages of C18:3n-6 and C18:3n-3 fatty acids in BF muscle compared with males. The detected differences in the composition of polyunsaturated fatty acids between the sexes are in agreement with the results found for farmed brown hares [26,30], but are in contrast with those for other wild species [47]. The hunting season highly affected PUFA composition, except for the oleic (C18:2n-6) fatty acid, in both muscles and α-linolenic (C18:3n-3) fatty acid in the BF muscle. Ruf et al. [53] have reported that linoleic (C18:2n-6) and arachidonic (C20:4n-6) acids of muscle phospholipids are positively related to the maximum running speed. These fatty acids were abundant in both muscles. Valencak et al. [55] reported the absence of pronounced differences in PUFA between skeletal muscle phospholipids from different location; however, those authors also reported that PUFA, including C20:4n-6, showed a seasonal winter increase compared with summer. Those authors also assumed that the high PUFA content found in the skeletal muscle in winter reflects thermoregulatory adjustments, e.g., regional heterothermy, to the severe climatic winter conditions in Central Europe. In Lithuania, the hunting of brown hares is allowable only in winter, and there is no possibility of comparing the seasonal variations of hare meat qualities. However, the percentage of C20:4n-6 in this study was considerably higher than that for wild hares in Spain [56] and farmed hares in Italy [30], although other authors [32] have found higher percentages of C20:4n-6 than in this study. 

Wild hare fat (Table 9) contained more SFA and MUFA, but slightly less PUFA, than IMF in muscles. Trace amounts of the lignoceric (C24:0) and nervonic (C24:1) fatty acids were determined only in the fat and not detected in the IMF of muscles. Although the percentages of most individual polyunsaturated fatty acids in the fat were lower than in the muscles, the percentage of the highly valuable C18:3n-3 acid in the fat was 2.12 and 2.45 times higher, respectively, than in the BF and LTL muscles. The ETA (C20:3n-3) acid was also 30% and 34.5% higher, respectively, than in the muscles. There were no differences in the percentages of total SFA, MUFA and PUFA in the fat between the sexes of the wild brown hare and their hunting seasons. Only the linoleic (C18:2n-6) fatty acid was lower (*p* < 0.01), and lauric (C12:0) acid and the trans isomer (C18:2t9,c12) of the C18:2 fatty acid were found to be higher (*p* < 0.05) in the fat of females compared with males.

Concerning saturated fatty acids, the hunting season affected (*p* < 0.01 and *p* < 0.001, respectively) the C12:0 and heneicosylic (C21:0) acids, whereas among MUFA, only the C15:1 and C17:1 fatty acids were affected (*p* < 0.05). Hunting season also had an effect (*p* < 0.01 and *p* < 0.05, respectively) on the polyunsaturated C24:6n-6 acid and the sum of EPA + DHA fatty acids.

### 3.5. Ratios of Fatty Acids and Lipid Quality Indices

The brown hare exhibited excellent PUFA/SFA and n-6/n-3 PUFA ratios in all the studied tissues (Table 10).

PUFA/SFA ratios were significantly above the minimum (0.4) and n-6/n-3 PUFA ratios were significantly lower than those recommended for the consumer diet [57,58]. Recommendations of Bellagio’s report on healthy agriculture, healthy nutrition and healthy people [58] emphasized that the n-6/n-3 PUFA ratio for a healthy diet should not exceed 4. The lowest n-6/n-3 PUFA ratio was found in the fat. The excellent n-6/n-3 PUFA ratios are in agreement with the findings of Valencak et al. [55,59], but in contrast with the findings for the farmed hare under controlled feeding, which showed a higher [27] and extremely undesirable (14.2–25.1) n-6/n-3 PUFA ratio [32]. The females demonstrated lower (*p* < 0.05 and *p* < 0.01, respectively) and more favourable n-6/n-3 PUFA ratios in the muscles and fat compared with males. The sex also appeared to affect the total proportion of total trans fatty acids (TFA). A higher (*p* < 0.01 and *p* < 0.05, respectively) percentage of TFA was found in the LTL and the fat of females. The females demonstrated a lower (*p* < 0.05) thrombogenic (TI) index in the LTL compared with males; however, no other more significant differences in the lipid quality indices between the sexes were found. The individual territory of a hare is up to 225 ha for males and up to 310 ha for females, and females change their location more often than males [17]. Females are more demanding on the environmental conditions during the birth and lactation of juveniles, and this can probably also affect their accumulated body deposits expressed as n-6/n-3 PUFA ratios and fat quality indices.

Hunting season affected (*p* < 0.001) only the peroxidizability index (PI) in both muscles and the proportions of unidentified fatty acids in all the studied tissues. The estimated values of atherogenic (AI) indices in both muscles were lower; however, the values of thrombogenic (TI) indices in the muscles were higher than those in the fat. TI indices determined in the present study for wild hare muscles were lower compared with the farmed hare [26,32] and wild hare [31] in different countries. In contrast, farmed hares in Italy [26] demonstrated lower, but Romania [31] and Poland [32] higher, AI indices than the free-living hares in the present study.

## 4. Conclusions

The sexual dimorphism of the wild brown hare has not been observed in either their carcass or internal organ traits. There were no pronounced differences found in other characteristics of meat chemical composition, physical–technological (colour, texture) properties and intramuscular fatty acid composition of both muscles. The hunting season showed an effect on the carcass weight and dorsal length. Smaller hares hunted during the first season had a higher percentage of dry matter, protein and hydroxyproline in both muscles but the values of pH, lightness (L*), drip loss and cooking loss were lower, whereas yellowness (b*) and hue angle (h) were higher compared with the larger hares hunted during the next hunting season. The hunting season also affected the shear force in both muscles and the toughness of the BF muscle. Significant differences in the levels of total MUFA and PUFA in both muscles were observed; however, there were no significant differences in the total SFA, MUFA and PUFA in fat between the hunting seasons. The brown hare exhibited excellent PUFA/SFA and n-6/n-3 PUFA ratios in all the studied tissues. 

The obtained results demonstrate the necessity for conservation and maintenance of the brown hare population, not only for biodiversity but also for the high-nutritional-value meat and lipids from this small game species.

## Figures and Tables

**Table 1:** Carcass measurements and muscularity in terms of thigh muscle-to-bone ratio between males and females and brown-hare hunting seasons.

Variables	Overall EM Mean *n* = 22	Sex		Hunting Season		SED	*p*-Value	
		Male *n* = 11	Female *n* = 11	I *n* = 12	II *n* = 10		Sex	HS
Carcass weight, g	2934.9	2866.1	3034.5	2770.8 ^c^	3129.8 ^d^	115.66	0.163	0.006
Dorsal length, cm	32.51	33.4	31.8	30.8 ^a^	34.4 ^b^	1.39	0.286	0.020
Thigh length, cm	9.77	9.9	9.6	9.6	9.9	0.47	0.457	0.500
Lumbar circumference, cm	21.07	20.7	21.4	21.2	20.9	0.44	0.116	0.419
Thigh weight, g	470.46	462.2	474.8	460.8	476.2	26.64	0.642	0.570
Muscles, g	376.68	371.5	379.3	371.0	379.8	17.07	0.652	0.615
Bones, g	93.77	90.7	95.5	89.8	96.5	13.08	0.721	0.615
Ratio M to B	4.31	4.46	4.16	4.42	4.19	0.490	0.552	0.640

SED—standard error of difference; HS—hunting season; *p* values of GLM LSD tests for hunting season were significantly different at ^a,b^ *p* < 0.05; ^c,d^ *p* < 0.01.

**Table 2:** Weight of internal organs of the brown hares.

Variables	Overall EM Mean *n* = 22	Sex		Hunting Season		SED	*p*-Value	
		Male *n* = 11	Female *n* = 11	I *n* = 12	II *n* = 10		Sex	HS
Liver, g	106.67	109.0	104.8	107.3	106.5	11.31	0.716	0.942
Heart, g	45.94	45.1	46.5	45.7	45.9	2.50	0.580	0.936
Kidneys, g	25.77	25.1	26.2	26.0	25.3	1.74	0.544	0.696
Lungs, g	74.69	68.2	71.1	54.3 ^a^	85.1 ^b^	12.94	0.827	0.038
Scapular fat, g	6.89	7.0	6.7	6.8	7.0	2.11	0.863	0.978
Internal fat	168.21	175.0	167.7	176.0	167.2	52.22	0.788	0.775

SED—standard error of difference; HS—hunting season; *p* values of GLM LSD tests for hunting season were significantly different at ^a,b^ *p* < 0.05.

**Table 3:** Effects of sex and hunting season on chemical composition of brown hare meat.

Variables	Overall Mean *n* = 22	Sex		Hunting Season		SED	*p*-Value	
		Male *n* = 11	Female *n* = 11	I *n* = 12	II *n* = 10		Sex	HS
*Longissimus thoracis et lumborum* (LTL)								
Dry matter, %	26.54	26.52	26.46	26.84	26.14	0.382	0.870	0.087
Protein, %	21.90	21.57	21.70	23.11 ^e^	20.16 ^f^	0.401	0.740	0.000
Fat, %	1.87	1.68	2.03	1.79	1.92	0.278	0.218	0.643
Ash, %	1.57	1.71	1.51	1.52	1.69	0.142	0.178	0.259
Hydroxyproline, mg/100 g	70.90	59.76	72.45	96.57 ^e^	35.64 ^f^	14.124	0.384	0.001
Cholesterol, mg/100 g	62.72	62.58	61.94	62.59	61.93	2.939	0.829	0.824
*Biceps femoris* (BF)								
Dry matter, %	25.95	25.25 ^a^	26.29 ^b^	26.35 ^a^	25.18 ^b^	0.464	0.039	0.021
Protein, %	21.58	21.23	21.24	22.53 ^e^	19.94 ^f^	0.545	0.988	0.000
Fat, %	2.07	1.81	2.30	2.17	2.30	0.405	0.240	0.563
Ash, %	1.41	1.42	1.46	1.38	1.50	0.139	0.740	0.411
Hydroxyproline, mg/100 g	87.36	68.51	92.00	110.60 ^c^	49.91 ^d^	17.79	0.204	0.003
Cholesterol, mg/100 g	60.91	60.41	58.91	62.58	56.75	3.257	0.652	0.095

SED—standard error of difference; HS—hunting season; *p* values of GLM LSD tests for sex and hunting season were significantly different at ^a,b^ *p* < 0.05; ^c,d^ *p* < 0.01; ^e,f^ *p* < 0.001; *p*-values of 0.05 ≤ *p* < 0.10 were considered trends.

**Table 4:** Effects of sex and hunting season on meat quality parameters of the brown hare.

Variables	Overall EM Mean *n* = 22	Sex		Hunting Season		SED	*p*-Value	
		Male *n* = 11	Female *n* = 11	I *n* = 12	II *n* = 10		Sex	HS
*Longissimus thoracis et lumborum* (LTL)								
pH	5.58	5.61	5.64	5.47 ^e^	5.78 ^f^	0.074	0.635	0.001
Colour L*	35.27	36.98	35.94	31.81 ^e^	41.11 ^f^	0.795	0.209	0.000
a*	20.51	20.74	20.32	20.10	20.96	0.798	0.603	0.295
b*	5.08	4.44	4.79	6.03 ^e^	3.20 ^f^	0.617	0.577	0.001
C	21.45	21.31	21.41	21.49	21.23	0.808	0.905	0.754
h	13.88	11.82	13.44	16.74 ^e^	8.51 ^f^	1.279	0.222	0.000
Drip Loss, %	1.66	2.18	1.96	0.79 ^e^	3.35 ^f^	0.373	0.559	0.000
Cooking loss, %	33.34	33.72	33.69	32.50 ^a^	34.91 ^b^	0.880	0.967	0.014
*Biceps femoris* (BF)								
pH	5.66	5.68	5.71	5.56 ^e^	5.82 ^f^	0.068	0.692	0.001
Colour L*	37.61	39.37	38.32	34.36 ^e^	43.32 ^f^	0.969	0.293	0.000
a*	20.77	20.82	20.70	20.72	20.80	0.720	0.875	0.904
b*	5.01	4.39	4.52	6.38 ^e^	2.54 ^f^	0.513	0.797	0.000
C	21.45	21.39	21.27	21.69	20.97	0.821	0.891	0.389
h	12.93	11.65	11.39	16.15 ^e^	6.89 ^f^	1.526	0.863	0.000
Drip Loss, %	1.00	1.58 ^a^	1.04 ^b^	0.33 ^e^	2.29 ^f^	0.251	0.044	0.000
Cooking loss, %	34.41	34.59	34.95	33.26	36.28	1.440	0.808	0.051

SED—standard error of difference; HS—hunting season; *p* values of GLM LSD tests for sex and hunting season were significantly different at ^a,b^ *p* < 0.05; ^e,f^ *p* < 0.001; *p*-values of 0.05 ≤ *p* < 0.10 were considered trends.

**Table 5:** Parameters of texture profile analysis (TPA) and Warner–Bratzler shear force of brown hare meat.

Variables	Overall EM Mean *n* = 22	Sex		Hunting Season		SED	*p*-Value	
		Male *n* = 11	Female *n* = 11	I *n* = 12	II *n* = 10		Sex	HS
*Longissimus thoracis et lumborum* (LTL)								
TPA: Cohesiveness	2.63	2.68	2.60	2.66	2.62	0.186	0.682	0.815
Gumminess, N	14.14	12.69	14.85	15.19	12.35	2.960	0.474	0.350
Hardness, N	35.49	32.62	37.44	37.95	32.11	5.560	0.397	0.307
Springiness	0.82	0.82	0.82	0.82	0.82	0.006	0.981	0.188
Chewiness, N	11.65	10.44	12.20	12.58	10.05	2.520	0.493	0.328
WB: Shear force, Nm	1.75	1.53	1.75	2.08 ^e^	1.20 ^f^	0.151	0.155	0.000
Toughness, N	105.06	58.82	152.97	74.53	137.26	83.72	0.276	0.463
*Biceps femoris* (BF)								
TPA: Cohesiveness	2.51	2.50	2.40	2.61 ^a^	2.29 ^b^	0.141	0.511	0.032
Gumminess, N	13.82	12.22	15.81	13.88	14.15	2.303	0.137	0.906
Hardness, N	34.31	30.61	37.73	35.49	32.85	5.535	0.214	0.640
Springiness	0.83	0.83	0.84	0.83	0.84	0.009	0.171	0.303
Chewiness, N	11.48	10.09	13.20	11.51	11.79	1.935	0.125	0.888
WB: Shear force, Nm	2.14	1.79	2.23	2.37 ^c^	1.65 ^d^	0.243	0.090	0.009
Toughness, N	697.56	517.52	636.45	986.44 ^e^	167.53 ^f^	82.19	0.165	0.000

SED—standard error of difference; HS—hunting season; *p* values of GLM LSD tests for sex and hunting season were significantly different at ^a,b^ *p* < 0.05; ^c,d^ *p* < 0.01; ^e,f^ *p* < 0.001; *p*-values of 0.05 ≤ *p* < 0.10 were considered trends.

**Table 6:** Effect of sex and hunting season on saturated fatty acid (% of total FA) composition in intramuscular fat of brown-hare muscles.

Muscle	Fatty Acids	Overall EM Mean *n* = 22	Sex		Hunting Season		SED	*p*-Value	
			Male *n* = 11	Female *n* = 11	I *n* = 12	II *n* = 10		Sex	HS
LTL	C12:0	0.06	0.06	0.06	0.05	0.08	0.015	0.595	0.060
	C14:0	1.82	1.85	2.03	1.56 ^c^	2.33 ^d^	0.265	0.507	0.009
	C15:0	0.21	0.19	0.23	0.22	0.20	0.030	0.139	0.486
	C16:0	21.23	20.98	22.19	20.48	22.68	1.275	0.355	0.101
	C17:0	0.36	0.33	0.36	0.37 ^a^	0.32 ^b^	0.024	0.165	0.023
	C18:0	7.97	7.49	7.58	8.66 ^e^	6.42 ^f^	0.588	0.889	0.001
	C20:0	0.05	0.04	0.05	0.05	0.04	0.016	0.732	0.713
	C21:0	0.13	0.12	0.14	0.11	0.16	0.030	0.490	0.160
	C22:0	0.04	0.04	0.04	0.04	0.05	0.015	0.999	0.495
	SFA	31.87	31.10	32.69	31.53	32.26	1.205	0.204	0.554
BF	C12:0	0.05	0.05	0.06	0.04	0.07	0.018	0.571	0.150
	C14:0	1.73	1.81	1.91	1.51 ^c^	2.22 ^d^	0.202	0.617	0.002
	C15:0	0.22	0.21	0.24	0.23	0.22	0.021	0.143	0.614
	C16:0	22.01	22.05	22.39	21.59	22.86	0.919	0.717	0.182
	C17:0	0.35	0.33	0.36	0.36	0.33	0.025	0.222	0.330
	C18:0	7.27	6.98	6.91	7.75 ^a^	6.14 ^b^	0.623	0.917	0.018
	C20:0	0.04	0.03	0.04	0.05 ^a^	0.02 ^b^	0.013	0.399	0.034
	C21:0	0.13	0.14	0.13	0.11	0.16	0.037	0.839	0.236
	C22:0	0.37	0.03	0.04	0.03	0.04	0.011	0.461	0.183
	SFA	31.85	31.63	32.09	31.66	32.06	0.844	0.592	0.645

LTL—*Longissimus thoracis et lumborum;* BF—*Biceps femoris;* SED—standard error of difference; HS—hunting season. *p* values of GLM LSD tests for hunting season were significantly different at ^a,b^ *p* < 0.05; ^c,d^ *p* < 0.01; ^e,f^ *p* < 0.001; *p*-values of 0.05 ≤ *p* < 0.10 were considered trends.

**Table 7:** Effect of sex and hunting season on monounsaturated fatty acid (% of total FA) composition in intramuscular fat of brown hare muscles.

Muscle	Fatty Acids	Overall EM Mean *n* = 22	Sex		Hunting Season		SED	*p*-Value	
			Male *n* = 11	Female *n* = 11	I *n* = 12	II *n* = 10		Sex	HS
LTL	C14:1n-7	0.20	0.22	0.22	0.18	0.26	0.048	0.954	0.094
	C15:1	0.13	0.12	0.11	0.16 ^a^	0.08 ^b^	0.035	0.742	0.031
	C16:1n9trans	0.08	0.08	0.08	0.08	0.08	0.023	0.894	0.833
	C16:1n-9	0.21	0.20	0.21	0.22	0.19	0.026	0.803	0.250
	C16:1n-7	4.71	5.40	4.82	4.10 ^a^	6.12 ^b^	0.778	0.469	0.018
	C17:1n-9	0.28	0.28	0.25	0.31	0.23	0.045	0.502	0.083
	C18:1n-9trans	0.57	0.50 ^a^	0.65 ^b^	0.56	0.59	0.052	0.012	0.515
	C18:1n-9	12.10	13.28	12.68	10.71 ^e^	15.25 ^f^	1.052	0.576	0.000
	C18:1n-7	1.11	1.22	1.08	1.06	1.24	0.108	0.218	0.102
	C20:1n-9	0.11	0.13	0.11	0.11	0.13	0.018	0.198	0.208
	MUFA	19.91	21.77	20.45	18.07 ^e^	24.16 ^f^	1.428	0.365	0.000
BF	C14:1n-7	0.19	0.22	0.19	0.17	0.23	0.034	0.430	0.091
	C15:1	0.12	0.11	0.11	0.14	0.08	0.038	0.975	0.140
	C16:1n9trans	0.08	0.08	0.08	0.07	0.09	0.016	0.575	0.391
	C16:1n-9	0.22	0.19	0.23	0.24 ^a^	0.18 ^b^	0.025	0.209	0.020
	C16:1n-7	5.19	5.86	5.13	4.82	6.18	0.795	0.370	0.103
	C17:1n-9	0.32	0.34	0.31	0.32	0.33	0.030	0.360	0.867
	C18:1n-9trans	0.53	0.52	0.59	0.50	0.61	0.067	0.294	0.148
	C18:1n-9	12.47	12.95	13.46	11.48 ^c^	14.93 ^d^	1.072	0.642	0.005
	C18:1n-7	1.16	1.20	1.19	1.13	1.26	0.147	0.907	0.397
	C20:1n-9	0.13	0.14	0.13	0.13	0.14	0.021	0.755	0.568
	MUFA	20.67	21.82	21.59	19.39 ^c^	24.01 ^d^	1.575	0.888	0.009

LTL—*Longissimus thoracis et lumborum;* BF—*Biceps femoris;* SED—standard error of difference; HS—hunting season. *p* values of GLM LSD tests for sex and hunting season were significantly different at ^a,b^ *p* < 0.05; ^c,d^ *p* < 0.01; ^e,f^ *p* < 0.001, and *p*-values of 0.05 ≤ *p* < 0.10 were considered trends.

**Table 8:** Effect of sex and hunting season on polyunsaturated fatty acid (% of total FA) composition in intramuscular fat of brown hare muscles.

Muscle	Fatty Acids	Overall EM Mean *n* = 22	Sex		Hunting Season		SED	*p*-Value	
			Male *n* = 11	Female *n* = 11	I *n* = 12	II *n* = 10		Sex	HS
LTL	C18:2n-6trans9,12	0.08	0.08	0.08	0.09 ^a^	0.06 ^b^	0.013	0.956	0.021
	C18:2n6t,c	0.10	0.04	0.13	0.00	0.09	0.064	0.214	-
	C18:2n-6	17.34	18.90	15.91	17.10	17.71	1.526	0.065	0.689
	C18:3n-6	0.12	0.12	0.12	0.13 ^a^	0.11 ^b^	0.011	0.783	0.029
	C18:3n-3	12.06	10.70 ^a^	13.81 ^b^	11.94	12.58	1.430	0.043	0.661
	C20:2n-6	0.17	0.18	0.15	0.19 ^e^	0.14 ^f^	0.013	0.081	0.001
	C20:3n-6	0.39	0.38	0.34	0.45 ^e^	0.28 ^f^	0.045	0.438	0.001
	C20:3n-3	0.29	0.24 ^a^	0.31 ^b^	0.33 ^e^	0.22 ^f^	0.025	0.014	0.001
	C20:4n-6	6.32	6.18	5.57	7.04 ^e^	4.72 ^f^	0.622	0.340	0.001
	C20:5n-3	1.94	1.57	1.90	2.33 ^e^	1.14 ^f^	0.232	0.171	0.000
	C22:2n-6	0.08	0.06	0.06	0.11 ^e^	0.01 ^f^	0.022	0.899	0.000
	C22:4n-6	0.12	0.12	0.10	0.15 ^e^	0.06 ^f^	0.014	0.186	0.000
	C22:5n-3	2.43	2.25	2.21	2.81 ^e^	1.64 ^f^	0.276	0.899	0.000
	C22:6n-3	0.48	0.46	0.46	0.54 ^a^	0.37 ^b^	0.069	0.998	0.021
	EPA + DHA	2.42	2.02	2.35	2.88 ^e^	1.50 ^f^	0.280	0.253	0.000
	PUFA	41.86	41.24	41.07	43.20 ^c^	39.11 ^d^	1.265	0.894	0.004
BF	C18:2n-6trans9,12	0.08	0.07	0.08	0.10 ^c^	0.05 ^d^	0.015	0.399	0.005
	C18:2n6t,c	0.10	0.04	0.14	0.00	0.09	0.067	0.206	-
	C18:2n-6	17.42	18.54	15.98	17.16	17.35	1.392	0.082	0.893
	C18:3n-6	0.12	0.10 ^c^	0.13 ^d^	0.13 ^e^	0.09 ^f^	0.007	0.003	0.000
	C18:3n-3	14.05	12.36 ^a^	16.32 ^b^	14.17	14.52	1.707	0.032	0.840
	C20:2n-6	0.17	0.17	0.17	0.19	0.14	0.015	0.970	0.004
	C20:3n-6	0.33	0.33	0.28	0.36 ^c^	0.25 ^d^	0.038	0.166	0.007
	C20:3n-3	0.30	0.26 ^a^	0.32 ^b^	0.33 ^c^	0.24 ^d^	0.024	0.018	0.002
	C20:4n-6	5.27	5.52	4.34	5.62 ^a^	4.24 ^b^	0.632	0.076	0.042
	C20:5n-3	1.45	1.25	1.37	1.67 ^e^	0.95 ^f^	0.184	0.531	0.001
	C22:2n-6	0.06	0.05	0.04	0.10 ^e^	2.26 ^f^	0.014	0.361	0.000
	C22:4n-6	0.12	0.11	0.10	0.15 ^e^	0.06 ^f^	0.018	0.580	0.000
	C22:5n-3	2.56	2.48	2.21	2.90 ^c^	1.79 ^d^	0.317	0.413	0.002
	C22:6n-3	0.50	0.46	0.45	0.55 ^a^	0.36 ^b^	0.078	0.925	0.023
	EPA + DHA	1.95	1.71	1.82	2.22 ^e^	1.31 ^f^	0.242	0.656	0.001
	PUFA	42.49	41.72	41.87	43.44 ^a^	40.15 ^b^	1.169	0.904	0.011

LTL—*Longissimus thoracis et lumborum;* BF—*Biceps femoris;* SED—standard error of difference; HS—hunting season. *p* values of GLM LSD tests for sex and hunting season were significantly different at ^a,b^ *p* < 0.05; ^c,d^ *p* < 0.01; ^e,f^ *p* < 0.001; *p*-values of 0.05 ≤ *p* < 0.10 were considered trends.

**Table 9:** Effect of sex and hunting season on fatty acid composition (% of total FA) of brown hare fat.

Fatty Acids	Overall EM Mean *n* = 22	Sex		Hunting Season		SED	*p*-Value	
		Male *n* = 11)	Female *n* = 11	I *n* = 12	II *n* = 10		Sex	HS
C12:0	0.09	0.08 ^a^	0.12 ^b^	0.07 ^c^	0.13 ^d^	0.021	0.049	0.009
C14:0	3.02	2.99	3.02	1.95	3.10	0.166	0.731	0.382
C15:0	0.36	0.34	0.34	0.40	0.29	0.054	0.985	0.068
C16:0	26.91	27.03	26.31	27.13	26.22	0.929	0.444	0.341
C17:0	0.44	0.42	0.45	0.45	0.43	0.032	0.352	0.579
C18:0	3.86	3.71	4.04	3.88	3.87	0.443	0.461	0.989
C20:0	0.07	0.08	0.07	0.08	0.06	0.013	0.580	0.057
C21:0	0.07	0.07	0.11	0.01 ^e^	0.17 ^f^	0.033	0.188	0.000
C22:0	0.02	0.01	0.02	0.02	0.01	0.007	0.118	0.062
C24:0	0.03	0.02	0.03	0.03	0.02	0.010	0.863	0.185
SFA	34.90	34.75	34.54	35.01	34.28	1.055	0.843	0.499
C14:1n-7	0.11	0.10	0.13	0.09	0.14	0.027	0.264	0.062
C15:1	0.03	0.03	0.05	0.00 ^a^	0.08 ^b^	0.034	0.590	0.027
C16:1n9trans	0.08	0.08	0.08	0.08	0.08	0.010	0.713	0.649
C16:1n-9	0.34	0.32	0.35	0.37	0.30	0.066	0.596	0.360
C16:1n-7	3.38	3.50	3.23	3.33	3.40	0.501	0.594	0.889
C17:1n-9	0.27	0.24	0.26	0.32 ^a^	0.18 ^b^	0.060	0.714	0.024
C18:1n-9trans	0.57	0.47	0.69	0.54	0.62	0.117	0.081	0.493
C18:1n-9	16.36	16.69	15.83	16.66	15.86	1.200	0.486	0.511
C18:1n-7	1.29	1.28	1.42	1.24	1.46	0.374	0.726	0.573
C20:1n-9	0.23	0.27	0.21	0.22	0.26	0.036	0.085	0.249
C24:1	0.04	0.03	0.04	0.04	0.03	0.008	0.207	0.296
MUFA	22.73	23.02	22.30	22.92	22.40	1.438	0.623	0.721
C18:2n-6trans9,12	0.07	0.08	0.07	0.08	0.07	0.014	0.539	0.376
C18:2n6c,t	0.03	0.02	0.04	0.00	0.03	0.017	0.299	-
C18:2n6t,c	0.13	0.09 ^a^	0.18 ^b^	0.11	0.16	0.040	0.038	0.250
C18:2n-6	10.66	12.67 ^c^	9.55 ^d^	10.18	12.04	0.927	0.003	0.060
C18:3n-6	0.12	0.13	0.12	0.12	0.13	0.015	0.751	0.218
C18:3n-3	29.78	27.64	31.43	30.12	28.95	1.919	0.064	0.552
C20:2n-6	0.13	0.14	0.13	0.13	0.14	0.016	0.537	0.606
C20:3n-6	0.13	0.02	0.01	0.02	0.01	0.007	0.064	0.668
C20:3n-3	0.39	0.35	0.42	0.42	0.34	0.051	0.200	0.149
C20:4n-6	0.09	0.13	0.08	0.05 ^c^	0.16 ^d^	0.032	0.134	0.003
C20:5n-3	0.06	0.05	0.08	0.04	0.09	0.028	0.316	0.087
C22:2n-6	0.02	0.03	0.02	0.03	0.02	0.008	0.161	0.874
C22:4n-6	0.02	0.01	0.04	0.02	0.03	0.019	0.175	0.618
C22:5n-3	0.11	0.12	0.11	0.11	0.13	0.017	0.365	0.207
C22:6n-3	0.003	0.004	0.004	0.00	0.008	0.004	0.912	0.075
EPA + DHA	0.06	0.05	0.08	0.04	0.10	0.027	0.311	0.045
PUFA	41.55	41.41	42.18	41.29	42.31	1.715	0.658	0.562

LTL—*Longissimus thoracis et lumborum;* BF—*Biceps femoris;* SED—standard error of difference; HS—hunting season; *p* values of GLM LSD tests for sex and hunting season were significantly different at ^a,b^ *p* < 0.05; ^c,d^ *p* < 0.01; ^e,f^ *p* < 0.001; *p*-values of 0.05 ≤ *p* < 0.10 were considered trends.

**Table 10:** Total trans fatty acids and fatty acid ratios and lipid quality indices in intramuscular and scapular fats from the brown hare.

Tissue	Variables	Overall EM Mean	Sex		Hunting Season		SED	*p*-Value	
			Male *n* = 11	Female *n* = 11	I *n* = 12	II *n* = 10		Sex	HS
LTL	TFA	0.77	0.68 ^c^	0.87 ^d^	0.73	0.81	0.062	0.006	0.195
	PUFA/SFA	1.33	1.34	1.27	1.38	1.23	0.079	0.431	0.073
	n-6/n-3	1.54	1.89 ^a^	1.25 ^b^	1.46	1.68	0.269	0.028	0.415
	AI	0.46	0.45	0.50	0.44	0.51	0.043	0.320	0.102
	TI	0.42	0.44	0.41	0.41	0.44	0.021	0.169	0.090
	h/H	2.45	2.50	2.32	2.55	2.27	0.218	0.431	0.227
	PI	99.76	94.37	96.89	107.56 ^e^	83.70 ^f^	4.658	0.595	0.000
	UFA	6.80	6.27	6.06	7.84 ^e^	4.49 ^f^	0.693	0.784	0.001
BF	TFA	0.73	0.69	0.83	0.68	0.84	0.080	0.093	0.060
	PUFA/SFA	1.34	1.33	1.31	1.38	1.26	0.060	0.784	0.064
	n-6/n-3	1.34	1.58 ^a^	1.08 ^b^	1.28	1.37	0.216	0.031	0.679
	AI	0.46	0.46	0.48	0.44	0.50	0.028	0.654	0.062
	TI	0.39	0.42 ^a^	0.37 ^b^	0.38	0.41	0.022	0.033	0.214
	h/H	2.39	2.37	2.34	2.45	2.27	0.149	0.841	0.233
	PI	97.56	94.11	93.89	102.96 ^e^	85.04 ^f^	4.458	0.961	0.001
	UFA	5.29	5.09	4.64	5.95 ^e^	3.78 ^f^	0.585	0.448	0.001
Fat	TFA	0.80	0.67 ^a^	0.98 ^b^	0.70	0.95	0.131	0.033	0.073
	PUFA/SFA	1.20	1.20	1.23	1.19	1.24	0.076	0.750	0.501
	n-6/n-3	0.39	0.50 ^c^	0.32 ^d^	0.36	0.46	0.056	0.005	0.096
	AI	0.61	0.61	0.60	0.61	0.60	0.031	0.794	0.756
	TI	0.31	0.33	0.29	0.31	0.31	0.023	0.113	0.964
	h/H	1.99	1.99	2.03	1.99	2.04	0.107	0.715	0.665
	PI	73.62	71.45	76.03	73.46	74.02	3.381	0.192	0.872
	UFA	0.82	0.78	0.95	0.72	1.01	0.068	0.021	0.001

LTL—*longissimus thoracis et lumborum*; BF—*biceps femoris.* TFA—sum of all identified trans fatty acids; PUFA/SFA—ratio of ΣPUFA to ΣSFA; n-6/n-3—ratio of Σn-6 PUFA to Σn-3 PUFA; AI—atherogenic index; TI—thrombogenic index; h/H—hypocholesterolemic/hypercholesterolemic ratio; PI—peroxidizability index; UFA—sum of unidentified fatty acids and their isomers. SED—standard error of difference; HS—hunting season. *p* values of GLM LSD tests for sex and hunting season were significantly different at at ^a,b^ *p* < 0.05; ^c,d^ *p* < 0.01; ^e,f^ *p* < 0.001; *p*-values of 0.05 ≤ *p* < 0.10 were considered trends.

## Data Availability

Data is contained within this article.
